# Development and Characterization of Effective Hemostatic Composites Based on Polyvinyl Alcohol/Kaolinite/Chitosan

**DOI:** 10.3390/polym17192637

**Published:** 2025-09-30

**Authors:** Aruzhan Alimbek, Bayansulu Otegenova, Zhanar Bekissanova, Balzhan Savdenbekova, Nailya Ibragimova, Renata Nemkayeva, Myroslav Sprynskyy, Alyiya Ospanova

**Affiliations:** 1 Faculty of Chemistry and Chemical Technology, Al-Farabi Kazakh National University, 050040 Almaty, Kazakhstan; aruzhan.alimbek5@gmail.com (A.A.); utegenova.bb@mail.ru (B.O.); bekisanova@gmail.com (Z.B.); balzhan.savdenbekova@gmail.com (B.S.); 2 Center of Physical-Chemical Methods of Research and Analysis, 050012 Almaty, Kazakhstan; 3 Scientific Center for Anti-Infectious Drugs, 050060 Almaty, Kazakhstan; nailya.73@mail.ru; 4 National Nanotechnology Laboratory, Al-Farabi Kazakh National University, 050040 Almaty, Kazakhstan; nemkayeva.renata@gmail.com; 5 Department of Environmental Chemistry and Bioanalytics, Faculty of Chemistry, Nicolaus Copernicus University in Torun, 7 Gagarina Str., 87-100 Torun, Poland; mspryn@umk.pl

**Keywords:** kaolinite, PVA, chitosan, hydrogel, hemocompatibility, wound healing

## Abstract

In this study, hemolytically safe and antibacterial polyvinyl alcohol/kaolinite/chitosan (PVA/KAO/CS) hydrogels were obtained using the freeze–thaw method. The structure of the chemical bonds present in the developed hydrogels was investigated by Fourier transform infrared spectroscopy (FTIR). Scanning electron microscopy (SEM) and optical microscopy study results showed the morphological and structural characteristics of the hydrogels’ surface. The thermal stability and phase transitions of the obtained hydrogel samples were determined by thermogravimetric analysis (TGA). Porosity, swelling, gel fractions, and mechanical properties were also examined. Biomedical properties of the samples were evaluated using in vitro and in vivo tests such as hemolytic activity, inhibition of protein denaturation, antimicrobial activity, and hemostatic activity. The obtained hydrogels demonstrated safe hemolytic activity, pronounced hemostatic activity, the ability to prevent thermal denaturation of albumin, as well as antimicrobial activity against Gram-positive bacteria *Staphylococcus aureus* ATCC BAA-39 and *Streptococcus pyogenes* ATCC 19615 and Gram-negative bacteria *Pseudomonas aeruginosa* ATCC 9027 and *Escherichia coli* ATCC 8739. All the obtained characteristics confirmed the promising biomedical applications of the obtained hydrogels.

## 1. Introduction

Hemorrhage is the cause of at least 40% of deaths following trauma and 27% of worldwide maternal deaths [1,2]. Bleeding leads to insufficient oxygen delivery for cellular metabolism and subsequent tissue hypoxia. This condition is also known as hemorrhagic shock [3,4]. Proper wound dressing mechanisms and the most acceptable dressing are essential for healing. This can be lifesaving, as wounds are a breeding ground for secondary pathogens that can cause wound suppuration and lead to death from sepsis [5,6]. Studies have indicated that inexpensive and effective hemostatic agents can bring economic benefits to hospitals and the medical industry [7,8]. The ideal hemostatic dressing should be accessible and inexpensive, simple to use, without side effects, consistent in various environmental conditions and have a long shelf life [9]. Hence, this perspective emphasizes the need to formulate new, stable, and reliable hemostatic agents [10].

A human blood coagulation system consists of a series of interconnected glycoproteins that, when activated, initiate the production of downstream enzymes and ultimately form fibrin [11]. In the instance of a wound, the clotting system quickly forms a blood clot. The estimated time to stop skin bleeding averages 2–5 min if the system is functioning properly [12]. When the blood clotting process is initiated, cells and membrane remnants react with clotting factors to form effective macromolecular complexes that promote the formation of fibrin molecules [13,14].

Historically, natural clay minerals have been used as inorganic hemostatic materials due to their superior biocompatibility, abundant availability, and cost-effectiveness [15]. Kaolinite (KAO) is a universal clay mineral that is commonly used for a variety of products such as ceramics, coatings, water treatment, pesticides, and catalysis substrates [16,17]. Recently, the application of kaolinite has been extended to the field of medicine as a potent material for exogenous blood coagulation [18]. Given that the negative charges on the surface of kaolin can significantly promote blood clotting, kaolin should exhibit hemostatic properties. In addition, kaolin can induce factor XII and influence platelet behavior to further trigger the blood coagulation cascade [19,20].

Chitosan (CS) is used to produce films, fibers, hydrogels, solutions, and composites for biomedical applications including hemostasis [21]. In the work, a multifunctional 3D-printed CS hydrogel strengthened with halloysite clay nanotubes (HNTs) has been developed, exhibiting hemostatic, antibacterial, and shape-adaptable properties [22]. Positively charged amino groups and negatively charged red blood cell (RBC) membranes, which form a strong hemostatic plug at the site of injury, promote hemostasis [23]. Also, chitosan plays an important role in platelet adhesion, activation, and aggregation by enhancing the glycoprotein complex (GPIIb/GPIIa) and mobilizing intracellular calcium ([Ca^2+^]i) [24]. Cellulose is the most abundant polysaccharide with good biocompatibility and water-retention capacity [25,26,27]. Carboxymethylcellulose (CMC) is a derivative of cellulose and solves the solubility problem [28,29]. CMC-based sponges are commonly used for wound dressing due to benefits such as low cost and good water retention [30,31,32].

Hemostatic agents with polymeric hydrogels are widely used in medical practice. Hydrogels are hydrophilic three-dimensional polymer-based structures with a high water absorption potential that bear a parallel resemblance to biological tissues [33]. Due to its good hydrophilicity, gel-forming properties, ease of processing, and high chemical resistance, polyvinyl alcohol (PVA) is often applied despite its synthetic nature [34,35,36]. The freeze–thaw method represents a unique mechanism involving phase separation into a PVA-rich phase and a water-rich phase, resulting in a change in the crystalline structure of the PVA matrix [37,38]. This expands its potential applications in pharmaceuticals and biomedicine [39,40]. Mahmoud Elsabahy et al. developed a combination of chitosan and kaolin that significantly improved hemostasis [41]. PVA hemostatic composites with different concentrations of kaolin modified with penicillin and streptomycin [42] or chitosan and hydroxyapatite [43], hydrogels PVA-CMC containing Inebrin [44], PVA-based sponges with the addition of cedar oil [45], and PVA/kaolinite sponges with marjoram essential oil [46] can be potential dressings for wound healing, especially bleeding. Damaged skin areas are centers of microbial accumulation and release a large amount of exudate, which hinders wound healing. Therefore, the biomedical agents with a large porous structure and antibacterial activity can promote rapid wound healing [6].

The aim of the present work was to synthesize hemostatic effective hydrogels based on polyvinyl alcohol and biodegradable chitosan and natural clay mineral kaolinite (PVA/kaolinite/CS), possessing synergistic effects in wound healing. Additionally, prepared composite (PVA/kaolinite/CS) was modified with sodium carboxymethylcellulose, which can be beneficial for managing wounds with low to moderate exudate. The hydrogels were examined for hemocompatibility, inhibition of protein denaturation, hemostatic activity, and antibacterial activity against Gram-positive bacteria *Staphylococcus aureus* ATCC BAA-39 and *Streptococcus pyogenes* ATCC 19615 and Gram-negative bacteria *Pseudomonas aeruginosa* ATCC 9027 and *Escherichia coli* ATCC 8739. The physicochemical properties of hydrogels were characterized in terms of thermal stability, morphology and structure, porosity, swelling, gel fractions, and mechanical properties.

## 2. Materials and Methods

### 2.1. Characteristics of Materials and Reagents

Natural kaolinite was obtained from the Alekseev clay deposit (Kokshetau, Kazakhstan) and processed by water dispersion, fractionation (d_p_ < 0.050 mm), and drying at 100 °C, as described in previous work [47]. PVA was obtained from Sigma Aldrich, USA (M_W_ ~145 kDa, 99.0–99.8% hydrolysis); low molecular weight chitosan (CS, M_W_ = 50–190 kDa, deacetylation degree 75–85%) was obtained from Sigma Aldrich, St. Louis, Missouri, 63,103 USA. Na-carboxymethyl cellulose was obtained from Sigma Aldrich, St. Louis, MO 63,103 USA (NaCMC, M_W_ = 700 kDa). Triple distilled water (TD water) was used in the preparation of all aqueous solutions.

### 2.2. Preparation of Composite Sponge’s Hydrogel

The PVA/KAO/CS composite hydrogels were developed using a freezing–thawing cycle technique [42,45]. Aqueous solutions of 5% PVA were prepared by dissolving pre-weighed quantities of PVA powder in TD water with heating at 90–105 °C for 2 h. Next, the 50 mL of PVA solutions were cooled to room temperature, and then different amounts of kaolinite (0.25, 0.5 g) and chitosan (0.25 g, which dissolved in a 20 mL solution of 1% acetic acid) were added with vigorous stirring at 25 °C. Afterwards, the mixtures were left for 6 h under stirring and ultrasonicated for 1 h to obtain homogeneous composite solutions. Then, the PVA/KAO/CS solutions were poured into plastic Petri dishes, which were further placed in a freezer (Haier Biomedical, model DW-30L278, Qingdao, China) at −15 °C for 18 h and then thawed at room temperature for 6 h. After 1 cycle, a solution of NaCMC (0.2% of 5 mL) was added, mixed for 1 h and the freezing and thawing cycles were continued. The freezing/thawing cycle was conducted ten times. The four different samples of hydrogels with varying amounts of kaolinite (0.25, 0.5 g) with and without the addition of NaCMC were prepared and labeled as PVA/KAO_0.25_/CS and PVA/KAO_0.5_/CS, PVA/KAO_0.25_/CS/NaCMC, and PVA/KAO_0.5_/CS/NaCMC, alongside PVA/CS as a control. The developed hydrogels were frozen in liquid nitrogen for 10 min and then lyophilized for 24 h (Shanghai Famo Machinery, model LG-03, Shanghai, China) at an average temperature and pressure of −40 °C and 0.009 Pa for further examinations. The samples were then soaked in TD water for 24 h, in order to remove the soluble parts. Water was changed two times, every two hours, in the first 4 h.

As a result of the freeze–thawing of polyvinyl alcohol, a physically crosslinked three-dimensional hydrogel network was formed, in the structure of which chitosan and kaolinite were intercalated. To enhance the moisture retention properties of the composition, sodium carboxymethylcellulose (NaCMC) molecules were incorporated into the hydrogel [30,31].

### 2.3. Study of the Physicochemical Properties

The chemical structures of the designed hydrogels were investigated using a Fourier transform infrared spectrophotometer after thoroughly mixing a weight of 5 mg of each hydrogel with potassium bromide (KBr) and then analyzed using a (Shimadzu 8400S, Kyoto, Japan) ranging from 400 to 4000 ${cm}^{-1}$. The morphological and structural characteristics of the hydrogels’ surfaces were characterized by scanning electron microscopy in combination with energy dispersive X-ray (EDX) analysis (SEM EDX, Quanta3D200i Dual system, FEI, Houston, TX, USA), with lyophilized fragments mounted on metal holders using carbon tape. The thermal stability and phase transitions of the obtained materials were analyzed in the temperature range of 30–1050 °C using TGA-DSC (Thermal Analysis Instruments SDT 2960, TA Instruments, Warszawa, Poland). The surface morphology of the hemostatic composites was examined using an optical microscope, Leica DM 6000 M (Leica, Sweden), with focus stacking techniques. The analysis was performed under different magnifications to assess structural characteristics and surface uniformity [48,49]. In the selected area of the sample, a series of photographs were taken with different focus depths (15–20 photos, depending on the height difference and the degree of roughness). Further, in the Helicon Focus program, the resulting photographs were combined into one 2D image. In addition, a 3D model was created from the same photos to visualize the surface relief.

In a swelling test, lyophilized hydrogels (0.3–0.6 g) were initially weighed to determine their dry mass and then immersed in falcon tubes containing 5 mL of TD water at room temperature. The intervals are every 10 min during the first hour and every subsequent hour; the samples were then blotted using filter papers, and reweighed. The procedure was repeated for days. The swelling percentages of hydrogels were calculated using Equation (1) as follows:

(1) $$\mathrm{Swelling}\;\mathrm{ratio}\;(\%)= [\left(W_{s}-W_{d}\right)/W_{d}]\times 100,$$

where (W_s_) is the weight of the swollen hydrogels, while (W_d_) refers to the weight of the dry sample.

The gel fraction of the crosslinked material was determined following a sequential process. The hydrogel was dried in a vacuum oven at 50 °C until complete moisture removal, and the initial dry weight (W_i_) was recorded. The sample was then immersed in distilled water for 24 h. After swelling, the hydrogel was dried again in a vacuum oven at 50 °C, and the new dry weight (W_e_) was measured. The gel fraction was calculated using the following equation:

(2) $$\mathrm{Gel}\;(\%)=(W_{e}/W_{i})\times 100$$

For porosity measurement, hydrogel membranes were dried at 50 °C for 2 h (W_1_), then immersed in absolute ethanol for 4 h to fill the pores. After blotting excess ethanol, samples were weighed again (W_2_). Porosity was calculated as follows:

(3) $$\mathrm{Porosity}\;(\%)= [\left(W_{2}-W_{1}\right)/\rho V]\times 100$$

where W_1_ is the dry sample mass, W_2_ is the mass after ethanol immersion, V is the hydrogel volume, and ρ is the density of absolute ethanol.

The mechanical properties of the lyophilized hydrogel samples were evaluated using a TA.XTplus tensile analyzer (Stable MicroSystems, Godalming, UK). The samples were prepared in a rectangular shape with dimensions of approximately 16 mm (width) × 15 mm (length) × 0.300 mm (thickness). Tensile strength tests were performed at a constant speed of 400 mm/min, and each sample was tested in triplicate.

### 2.4. Study of the Biomedical Properties

All animal experiments were conducted after ethical approval by the Scientific Centre for Anti-Infectious Drugs (№ 04-03/390), and studies were carried out at the Scientific Center for Anti-Infectious Drugs.

To evaluate the hemocompatibility of the developed hydrogels hemolysis tests were performed according to the described methodology from [46] with slight adaptations. Blood with anticoagulant was prepared by adding 9 mL of blood obtained from a healthy volunteer into a tube with 1 mL of K_3_− EDTA, centrifuged at 3000 rpm for 10 min. The tubes were incubated at 37 °C for 1 h. Before contact with blood, 1 cm^2^ membrane samples were pre-immersed in phosphate buffer (PBS, pH 7.0) at 37 °C for 72 h. After incubation, PBS was removed, and the membranes were immersed in 1 mL of K_3_− EDTA − blood and left at 37 °C for 1 h. Then, the reaction mixture was centrifuged for 10 min at 1500 rpm at room temperature. The amount of hemoglobin released by hemolysis was determined using a spectrophotometer (BioMate 3S, 540 nm). Control Samples:

Negative control—50 mL of EDTA-anticoagulated blood+ 2 mL of PBS.

Positive control—50 mL of EDTA-anticoagulated blood + 2 mL of distilled water.

The experiment was performed three times, and the percentage of hemolysis was calculated using the following formula:

(4) $$\mathrm{Hemolysis}\;(\%):\;=\; [\left({OD}_{s}-{OD}_{n}\right)/{OD}_{P}-{OD}_{n}]\times 100$$

where ODs is the optical density of a tested sample, ODp is the optical density of the positive control, and ODn refers to the optical density of the negative control.

The test sample for the inhibition of protein denaturation was prepared by mixing 0.12 mL of egg albumin, 1.78 mL of phosphate-buffered solution (PBS, pH 7.0), and various concentrations of the test substance: 400 µg/mL and 600 µg/mL. Distilled water (2.0 mL) was used as the negative control. Dexamethasone at a final concentration of 100 µg/mL (2 mL) was used as the positive control. All samples were incubated at 37 °C for 15 min, followed by heating at 70 °C for another 15 min. After cooling to room temperature, the samples were centrifuged at 2500 rpm for 15 min. Turbidity was measured at 660 nm, and the percentage of inhibition was calculated using the following formula:

(5) $$\mathrm{Inhibition}\;(\%)= [\left({OD}_{s}-{OD}_{n}\right)/{OD}_{P}-{OD}_{n}]\times 100$$

where OD_s_ is the optical density of a tested sample, OD_p_ is the optical density of the positive control, and OD_n_ refers to the optical density of the negative control.

The hemostatic activity of the samples was evaluated in vivo using a liver injury model in white, outbred Wistar laboratory rats (male and female, *n* = 9, weight 250 g ± 10%.

The bleeding model was reproduced by laparotomy with a longitudinal incision along the white line of the abdomen, through which the anterior surface of the liver was exposed. Then, a standard parenchymatous wound was formed using a Dermo-Punch instrument with a diameter of 5 mm and a depth of 2 mm. After capillary-parenchymatous bleeding occurred, the test samples were applied to the damaged area. The agent completely covered the wound surface.

A commercially available hemostatic drug applied in a similar amount was used as a comparative substance. The time to stop bleeding was measured with a stopwatch, starting from the moment the sample was applied until the bleeding stopped completely. The criterion for stopping was the complete absence of visible blood penetration on the outer surface of the gauze swab. The volume of blood loss (P) was calculated based on the difference in the mass of the gauze swab before and after interaction with the wound surface using the following formula:

(6) $$P=M_{1}-M_{0}$$

where M_0_ is the initial weight of the dry gauze (mg), and M_1_ is the weight of the blood-soaked gauze (mg).

At the end of the experiment, the animals were euthanized, and biomaterials were collected. The study included the acclimatization of the animals, anesthesia, modeling of damage, application of materials, measurement of hemostasis parameters, and data collection.

The antimicrobial activity of the obtained hydrogels was investigated by diffusion test in dense Mueller–Hinton agar. Mueller–Hinton agar (MHA) is recommended by EUCAST and CLSI for the antimicrobial susceptibility diffusion assay (AST). Two Gram-positive (*Staphylococcus aureus* ATCC BAA-39, *Streptococcus pyogenes* ATCC 19615) and two Gram-negative (*Pseudomonas aeruginosa* ATCC 9027, *Escherichia coli* ATCC 8739) bacterial strains were selected as test strains. All of the test strains were obtained from the American Type Culture Collection (ATCC). The inoculum for each test strain was prepared by the direct colony method: an aliquot of the test strain was taken with a bacteriological loop, transferred to a tube with sterile saline and thoroughly homogenized to obtain a homogeneous suspension. The density of the suspension of each strain tested was 0.5 McFarland, confirmed spectrophotometrically (DEN-1, Biosan, Riga, Latvia), corresponding to ~1.5 × 10^8^ CFU/mL. Petri dishes with Mueller–Hinton agar were inoculated with the tested suspension. Sterile cotton swabs were used for inoculation, which were dipped in the bacterial suspension, then lightly pressed against the walls of the test tube, shaded in three directions, turning the Petri dish by 60°. Then, 6 mm diameter wells were made in the agar and 200 µL of each sample was added to the corresponding wells. The plates were placed at room temperature for 30 min to allow the samples to diffuse into the agar.

The plates were incubated in an incubator at 37 ± 1 °C for 24 h to study the zone of inhibition. All experiments were performed in triplicate and results were expressed as mean ± standard deviation using Excel program. Antibacterial activity was determined by measuring the diameter of the zones of inhibition in millimeters (mm) around the wells using the antibiotic zone scale.

## 3. Results

In the last decade, hydrogels based on many high molecular weight compounds and, in particular, polyvinyl alcohol have been successfully used in medical practice [35,36]. The use of polyvinyl alcohol and its derivatives is associated with their chemical and biological properties, such as biocompatibility, biodegradability, and environmental safety for the human body [50]. The main advantage of producing PVA-based hydrogels is the use of cyclic freeze–thaw method for the formation of crystalline clusters as a drug carrier [51]. The combination of chitosan, sodium carboxymethylcellulose (CMC), and kaolinite in a crosslinked hydrogel network allows for a promising material with a unique set of synergistic properties and a multifunctional effect that promotes wound healing to be obtained.

### 3.1. SEM Analysis of PVA/KAO/CS Composites

**Figure 1:**
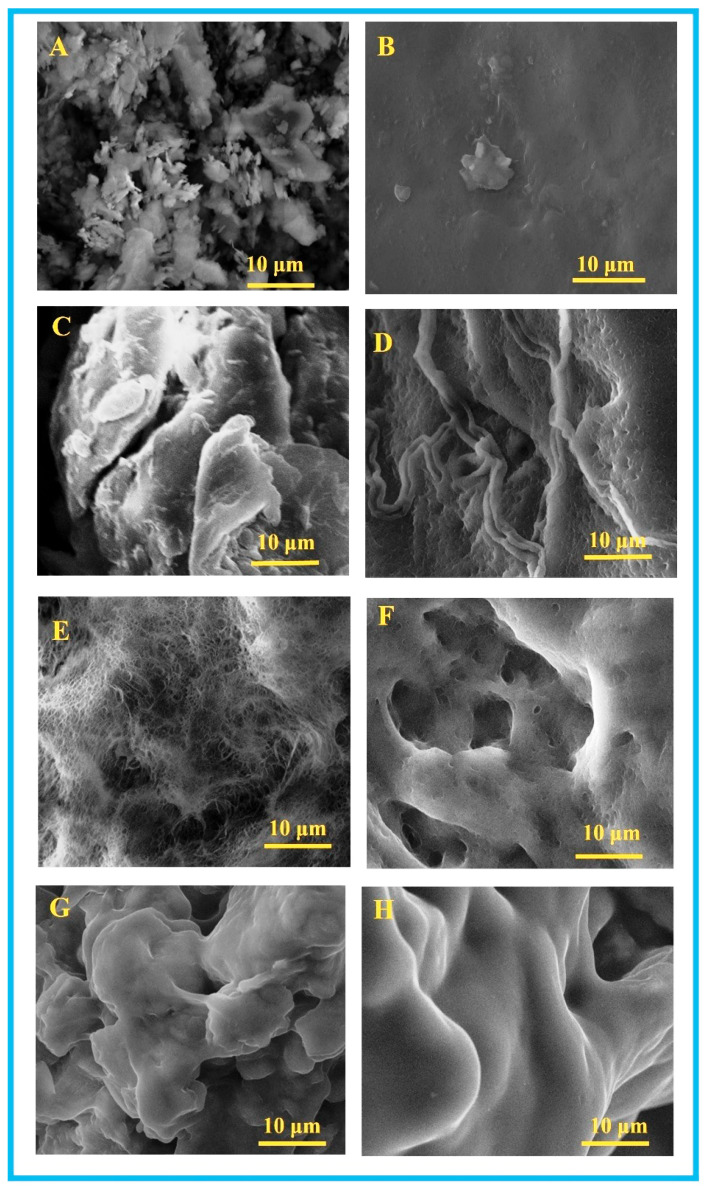
SEM images of Nat KAO (**A**), PVA (**B**), CS (**C**), PVA/CS (**D**), PVA/KAO_0.25_/CS (**E**), PVA/KAO_0.5_/CS (**F**), PVA/KAO_0.25_/CS/NaCMC (**G**), and PVA/KAO_0.5_/CS/NaCMC (**H**).

Scanning electron microscopy investigations determined the structure and morphology of natural kaolinite, PVA, CS, PVA/CS, and composites PVA/KAO_0.25_/CS, PVA/KAO_0.5_/CS, PVA/KAO_0.25_/CS/NaCMC, and PVA/KAO_0.5_/CS/NaCMC (Figure 1).

Morphological analyses of the initial reagents and their interaction products indicate significant differences in their SEM images. The microphotographs in Figure 1A show the morphology of natural kaolinite, which is characterized by a “sheet-like” structure of particles interconnected into denser structures [52]. The surface of pure PVA hydrogel (Figure 1B) is characterized by a more even surface and much less roughness and macroporosity compared with its hydrogels based on PVA/KAO/CS (Figure D–H). SEM images show significant morphological changes in the PVA/KAO/CS and PVA/KAO/CS/NaCMC composite hydrogels compared with the initial PVA/CS hydrogel (Figure 1D). The three-dimensional structure with the presence of pores and heterogeneity of the rough surface of PVA/KS is clearly visible, as well as the morphological differences for PVA/KAO/CS hydrogels with different kaolin concentrations. All PVA/KAO/CS hydrogels are characterized by a sponge-like structure consisting of interconnected macropores of irregular shape and varying sizes. The addition of kaolinite promotes the formation of a rigid porous microstructure of hydrogels. Large pores with a smooth surface of walls are visible in the PVA/KAO_0.5_/CS sample (Figure 1F), which also indicates the interaction of the polymer chains of PVA and chitosan with kaolin particles and the structural stability of this hydrogel. The addition of NaCMC to the hydrogel structure smoothes the surface and also reduces the macroporosity of the PVA/KAO_0.25_/CS/NaCMC hydrogel (Figure 1G) and PVA/KAO_0.5_/CS/NaCMC (Figure 1H).

**Figure 2:**
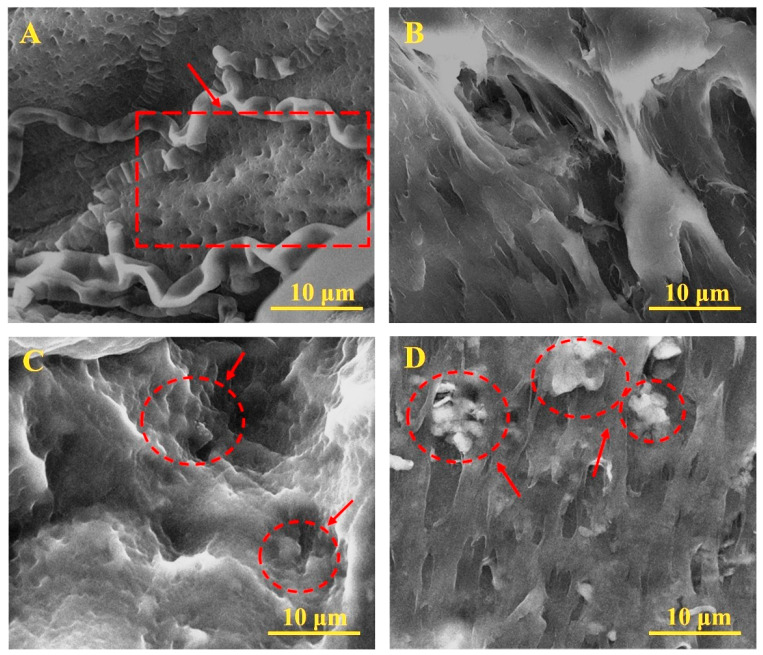
SEM cross section of images PVA/CS (**A**), PVA/CS/NaCMC (**B**), PVA/KAO_0.25_/CS/NaCMC (**C**), and PVA/KAO_0.5_/CS/NaCMC (**D**).

The SEM images of the cross sections of the hydrogels (Figure 2) show that in the PVA/CS structure (Figure 2A), pores of approximately equal size, characteristic of PVA hydrogel skeletons and chitosan molecule embeddings, are clearly visible. The addition of NaCMC molecules changes the hydrogel structure in the PVA/CS/NaCMC composition (Figure 2B); the small pores disappear and change to larger cavities. The hydrogels of PVA/KAO_0.25_/CS/NaCMC and PVA/KAO_0.5_/CS/NaCMC (Figure 2C,D) are strongly crosslinked porous formations with kaolin particles embedded in the structure, especially in the SEM image of the PVA/KAO_0.5_/CS/NaCMC sample. The hydrogels obtained were rougher (optical images) due to the presence of kaolin particles compared with unmodified PVA, with kaolin aggregation in the structure becoming greater with increasing kaolin concentration. The small concentrations of kaolin in PVA/KAO_0.25_/CS/NaCMC are probably bound uniformly to the PVA backbone purely electrostatically, and when the clay concentration increases, some of the KAO particles are distributed on the free areas of PVA due to adsorption forces on the active centers of microcracks along the polymer mesh, which is related to the rearrangement of particles within the three-dimensional hydrogel structure. The obtained results support the conclusions reported by Pourshahrestani, S et al. [23].

### 3.2. FTIR Analysis PVA/KAO/CS Hydrogels

**Figure 3:**
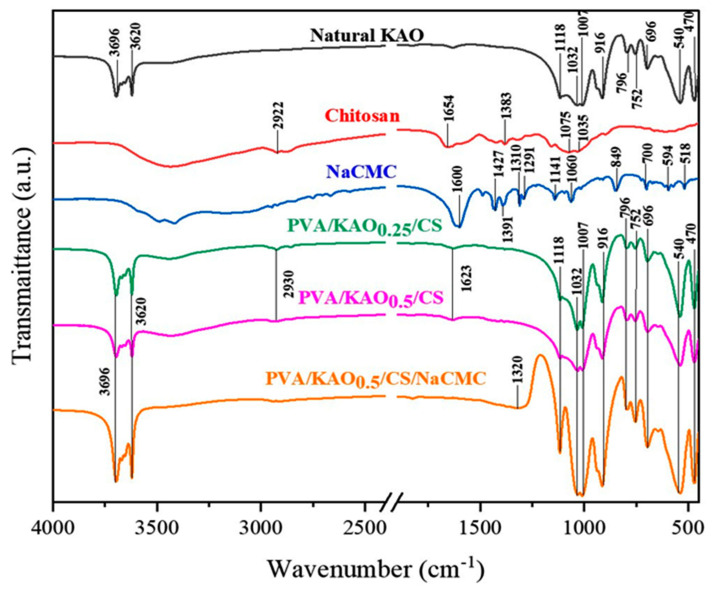
FTIR spectra of Nat KAO, chitosan, NaCMC, PVA/KAO_0.25_/CS, PVA/KAO_0.5_/CS, PVA/KAO_0.25_/CS/NaCMC, and PVA/KAO_0.5_/CS/NaCMC.

The functional groups in the natural kaolinite and hydrogels were characterized by FTIR (Figure 3). The FTIR spectrum of the kaolinite exhibits a strong the peaks at 1032 and 1118 cm^−1^ belonging to the Si-O vibration band; the peaks at 540, 752, and 796 cm^−1^ are attributed to the vibration bands of Si-O-Al bonds; the peak at 916 cm^−1^ is attributed to the Al-OH vibrations, and the peaks at 3696 and 3620 cm^−1^ belong to the stretching vibrations of the OH-groups [53,54].

The band at 2930 cm^−1^ corresponds to the stretching vibrations of C-H bonds of pure chitosan [55]. The band at 1654 cm^−1^ is associated with the C=O stretching vibration of the carbonyl group (amide I) in the chitosan structure [56]. This band was shifted to 1623 cm^−1^ in the PVA/KAO/CS biocomposite hydrogels. The reason for this frequency shift may be due to the electrostatic interaction between the protonated amino groups (NH^3+^) of chitosan and the negatively charged sites of kaolin clay [57,58]. This phenomenon confirms that an ion exchange reaction occurred between chitosan and kaolin, resulting in the intercalation of chitosan into the kaolin structure.

The disappearance of the peak at 1320 cm^−1^ belonging to the COO- vibrations of the carboxylate groups of pure NaCMC [59] in the PVA/KAO/CS biocomposite hydrogels may also be related to the interaction of NaCMC with kaolinite. The disappearance of the 2930 cm^−1^ (C-H) and 1623 cm^−1^ (C=O or NH_2_) peaks belonging to NaCMC [60] indicates a strong interaction between the carboxylate groups of NaCMC and the amino groups of chitosan.

### 3.3. Mechanical Properties of PVA/KAO/CS Hydrogels

**Figure 4:**
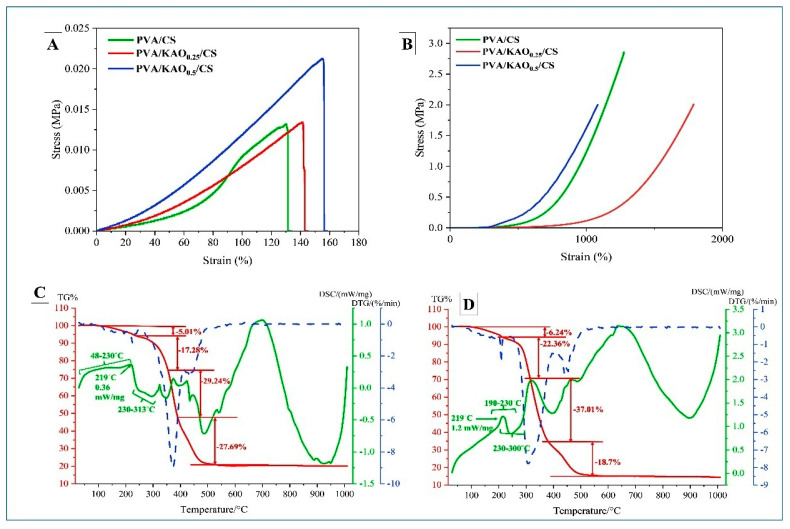
Stress–strain test (**A**) and compression test (**B**) of PVA/CS, PVA/KAO_0.25_/CS, PVA/KAO_0.5_/CS, TGA and DSC spectrum of PVA/KAO_0.25_/CS (**C**) and PVA/KAO_0.5_/CS (**D**).

The mechanical properties of hydrogels PVA/KAO_0.25_/CS and PVA/KAO_0.5_/CS are presented in Figure 4A,B. The tensile strength of PVA/KAO_0.5_/CS was 154.2%, higher than that of PVA/KAO_0.25_/CS, which was 141.3%. This result indicates that increasing kaolinite concentration improves both the tensile strength and elasticity of the material. The system can act as a reinforcing component, improving tensile strength. Kaolinite particles can fill the defects in the hydrogel structure, prevent the development of microcracks, and maintain the structure of the material, which makes it stronger and less prone to failure under tensile loads [61,62,63].

In the compression test, the stiffness value for the PVA/KAO_0.5_/CS hydrogel was 1082.1%, and its lower value compared with the other samples may indicate that a higher kaolinite content makes the material less stiff in compression. This may be because as the clay concentration increases, kaolinite particles disrupt the matrix structure, resulting in the formation of localized weak zones at microcrack junctions for compressive forces despite the material being stiffened in tension. This PVA/KAO_0.5_/CS hydrogel shows a higher strain value; it is a more compressive ductile and can deform more strongly before failure.

While kaolinite improves the tensile strength of the material by strengthening the structure in the PVA/KAO_0.5_/CS hydrogel, in compression tests it can, on the contrary, negatively affect the integrity of the material due to particle aggregation or disruption of the polymer matrix structure in the PVA/KAO_0.5_/CS hydrogel. This results in reduced resistance to compressive forces [64].

### 3.4. TGA of PVA/KAO/CS Hydrogels

The result of the TGA of PVA/KAO/CS hydrogels with different amounts of kaolinite is shown in Figure 4C,D. The thermographic curves show several stages of degradation of the obtained hydrogels. The first mass loss for PVA/KAO_0.25_/CS hydrogel was observed between 113 and 228 °C, in a quantity of 5.01%. For the PVA/KAO_0.5_/CS hydrogel, an initial decrease in mass was observed between 109 and 224 °C, in a quantity of 6.24%. This is probably due to the evaporation of water retained by hydrophilic hydroxyl groups in the polymer matrix [42]. The second pronounced mass loss of 17.28% in the temperature range from 228 to 348 °C for PVA/KAO_0.25_/CS and mass loss of 22.36% for PVA/KAO_0.5_/CS in the temperature range from 224 to 314 °C can be attributed to the removal of hydroxyl groups and formation of polyene macromolecules [65]. For PVA/KAO_0.25_/CS hydrogel, a mass loss of 29.24% was observed in the temperature range of 348–388 °C and a mass loss of 27.69% in the temperature range of 388–543 °C, while the PVA/KAO_0.5_/CS hydrogel recorded a mass loss of 37.01% in the temperature range of 314–384 °C and also a mass loss of 18.7% in the temperature range of 384–544 °C. The mass loss in both cases for both hydrogels corresponds to the thermal decomposition of the main PVA chain and the removal of water and carbon dioxide [46] and can be attributed to the decomposition of the organic residues of chitosan.

The residual mass at above 604 °C for PVA/KAO_0.25_/CS and the residual mass at above 598 °C for the PVA/KAO_0.5_/CS hydrogel refers to inorganic kaolinite residues that have not undergone decomposition at this temperature [46].

As shown in the DSC curves of PVA/KAO_0.25_/CS hydrogel, the exothermic peaks exhibited in the temperature range of 48–230 °C and the exothermic peaks for PVA/KAO_0.5_/CS exhibited in the temperature range of 190–230 °C can be attributed to relaxation associated with crystalline regions in the hydrogel [59]. The endothermic peaks at 230–313 °C and 230–300 °C for PVA/KAO_0.25_/CS and PVA/KAO_0.5_/CS, respectively, indicate the melting of PVA and the destruction of its crystal structure (Tm). In addition, changes in the Tm value for hydrogel towards higher temperatures indicate crosslinking of PVA and kaolin affecting crystal formation [66]. The subsequent exothermic peaks are due to the thermal degradation of PVA, chitosan, and the loss of water molecules along the main chain of the polymer. The effect of temperature on the stability of PVA/KAO_0.25_/CS/NaCMC and PVA/KAO_0.5_/CS/NaCMC hydrogels is almost similar.

### 3.5. Optical Microscopy of PVA/KAO/CS Hydrogels

**Figure 5:**
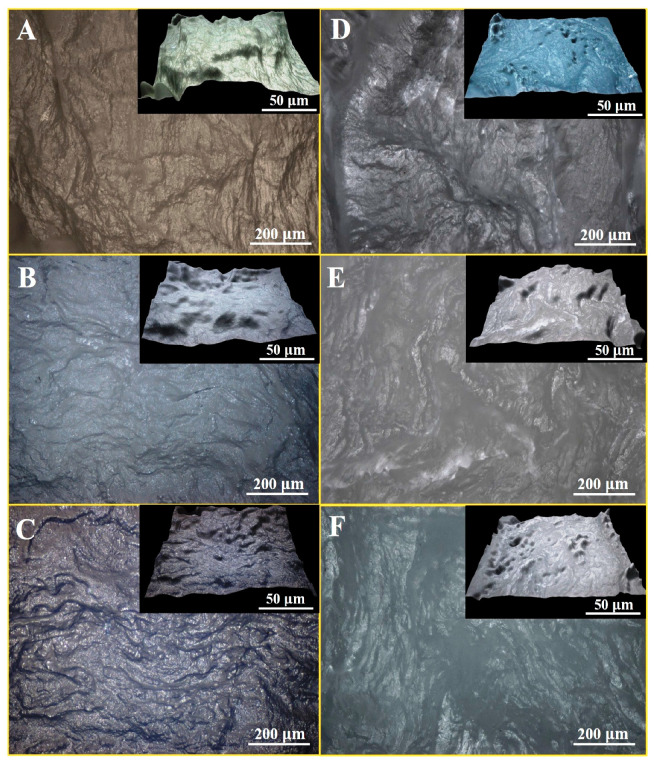
Optical microscopy images of PVA/CS (**A**), PVA/KAO_0.25_/CS (**B**), PVA/KAO_0.5_/CS (**C**), PVA/CS/NaCMC (**D**), PVA/KAO_0.25_/CS/NaCMC (**E**), and PVA/KAO_0.5_/CS/NaCMC (**F**).

The optical microscopy images of PVA/KAO/CS hydrogels with different amounts of kaolinite are presented in Figure 5. The analysis of optical images of the PVA-based hydrogels shows an irregular rough surface of the hydrogels, which are characterized by bulges of different sizes, especially distinct in the PVA/CS dual system (Figure 5A), the addition of kaolinite (Figure 5B,C) slightly smooths the structure; however, the kaolinite-based hemostatic membranes: PVA/KAO_0.25_/CS/NaCMC (Figure 5E) and PVA/KAO_0.5_/CS/NaCMC (Figure 5F) are characterized by a pronounced irregular surface. This confirms the spongy structure of the samples and suggests increased porosity and friability of the inner cavities of the hydrogels.

### 3.6. Gel Fraction, Porosity, Swelling Test of PVA/KAO/CS Hydrogels

**Figure 6:**
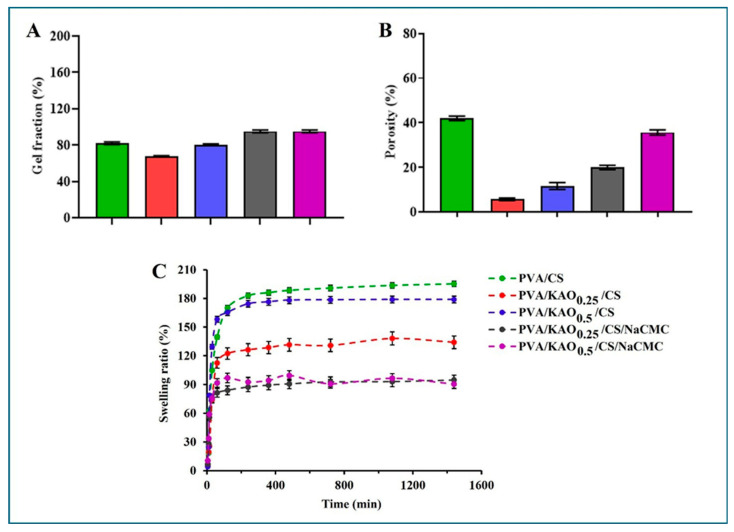
Gel fraction (**A**), porosity (**B**), and swelling ratio results (**C**) of samples of the obtained hydrogels PVA/CS, PVA/KAO_0.25_/CS, PVA/KAO_0.5_/CS, PVA/KAO_0.25_/CS/NaCMC, and PVA/KAO_0.5_/CS/NaCMC. The data are represented as means ± SD (n = 3).

The results of the gel fractions of the obtained hydrogels are presented in Figure 6A. The values were calculated according to Equation (2). The gel fraction of PVA/CS is about 80%; the addition of kaolin slightly decreases this parameter for PVA/KAO_0.25_/CS and PVA/KAO_0.5_/CS. This is probably because kaolin partially reduces the crosslinking of pure PVA. For the obtained PVA/KAO_0.5_/CS hydrogels, the gel fraction is 80 ± 0.71%, while those for PVA/KAO_0.25_/CS/NaCMC and PVA/KAO_0.5_/CS/NaCMC are 95 ± 1.41% and 97 ± 1.32%, respectively, indicating almost fully crosslinked hydrogels. The obtained values indicate that the incorporation of NaCMC in addition to kaolin and chitosan enhanced the crosslinking density and thereby increased the strength of the hydrogel. PVA/KAO_0.25_/CS/NaCMC and PVA/KAO_0.5_/CS/NaCMC gels could absorb excess wound exudate well compared with other samples.

The values of the hydrogels’ porosities (Figure 6B) were calculated according to Equation (3). An important characteristic of PVA is the formation of three-dimensional porous frameworks, which are additionally completed due to the introduced bioactive agents, and this changes their porosity. In [42], the authors found that the pore size of PVA/kaolin hydrogels was smaller than that of pure PVA, which led to a decrease in the crosslinking density of hydrogels with increasing kaolin content in the PVA matrix. The porosities of the PVA/KAO_0.25_/CS and PVA/KAO_0.5_/CS hydrogel is not large, and the porosity is 20 ± 1.35% for PVA/KAO_0.25_/CS/NaCMC hydrogel and 36 ± 1.45% for PVA/KAO_0.5_/CS/NaCMC hydrogel.

An important requirement for hemostatic agents is the contact of the swelling factor with bleeding during wound healing and good adsorption of the biological fluid [67]. Therefore, the obtained hydrogels PVA/KAO_0.25_/CS, PVA/KAO_0.5_/CS, PVA/KAO_0.25_/CS/NaCMC, and PVA/KAO_0.5_/CS/NaCMC are expected to exhibit the ability to absorb sufficient amounts of water or biological fluid from the wound surface; Figure 6C. The values were calculated according to Equation (1). The results of these studies showed that all samples absorb moisture well and swelling equilibrium is reached for almost all hydrogels within 60 min. The swelling capacities of the PVA/KAO_0.25_/CS/NaCMC and PVA/KAO_0.5_/CS/NaCMC hydrogels are almost the same and ranges from 75 to 94%; for PVA/KAO_0.25_/CS this parameter is slightly higher and ranges from 122 to 134%, and PVA/KAO_0.5_/CS is characterized by 174–179% absorption. These parameters are in agreement with the porosity results. It is known from the literature [68,69,70] that with similar swelling percentages, biomedical dressings have shown potential for their use in wound healing. It follows that the obtained hydrogels can also be promising bases for hemostatic dressings.

### 3.7. Biomedical Evaluations of PVA/KAO/CS Hydrogels

#### 3.7.1. Hemolytic Activity, Inhibition of Protein Denaturation, and Hemostatic Activity

**Figure 7:**
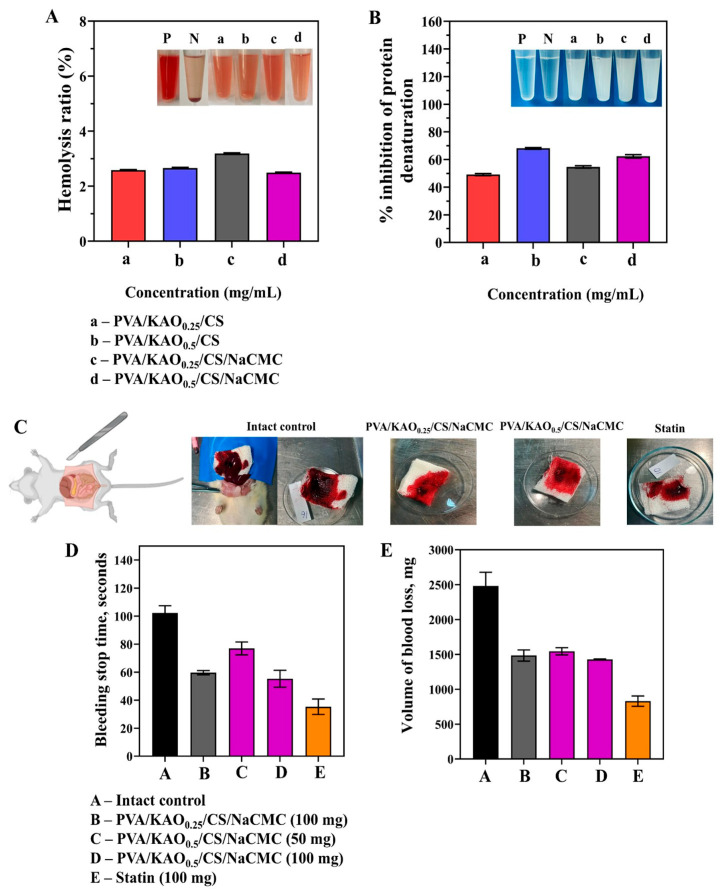
Hemolytic activity (**A**) and inhibition of protein denaturation (**B**) results of samples of the obtained hydrogels PVA/CS, PVA/KAO_0.25_/CS, PVA/KAO_0.5_/CS, PVA/KAO_0.25_/CS/NaCMC, and PVA/KAO_0.5_/CS/NaCMC. The data are represented as means ± SD (n = 2). Representative photographs of animal groups (**C**), results of average time to stop bleeding (seconds) in laboratory rats after application of the test substances (**D**), and volume of blood loss (mg) when simulating liver injury in rats (**E**). (M ± m). The data are presented as mean ± SD (n = 3).

When obtaining medical hydrogels, the compatibility of their compositions with blood is a prerequisite. In this regard, the obtained hydrogels were tested for hemolytic compatibility and the percentage of hemolysis of the studied samples is presented in Figure 7A. The values were calculated according to Equation (4). The results showed no significant differences in these values between the hydrogels and demonstrated a small hemolysis of about 2.5% and about 3% for PVA/KAO_0.25_/CS/NaCMC, which is considered a safe level according to ASTM [71]. In comparison with the results of hemolytic activity reported in this study [40], the obtained values were below 2%, which is also considered a safe level.

An important indicator of biomedical performance is the % inhibition of protein denaturation. The denaturation of protein is associated with the occurrence of inflammatory diseases such as rheumatoid arthritis, diabetes, cancer, etc. The substance’s ability to prevent protein denaturation may therefore also contribute to the prevention of inflammatory diseases [72].

All the samples showed different degrees of denaturation inhibition depending on their composition and concentration (µg/mL) (Figure 7B). The values were calculated according to Equation (5). The sample PVA/KAO_0.5_/CS showed the highest activity at a concentration of 600 µg/mL with an inhibition degree of 67.98 ± 0.39%. This may be due to the optimal ratio of components, at which a favorable polymer network is formed that prevents protein denaturation. The sample PVA/KAO_0.25_/CS demonstrated 48.96 ± 0.55% inhibition at 400 µg/mL concentration. In the PVA/KAO_0.25_/CS/NaCMC sample at a concentration of 400 µg/mL, the degree of inhibition was 55.12 ± 0.24%, which was the highest value among samples with lower kaolinite content. In the sample PVA/KAO_0.5_/CS/NaCMC, it was observed that at a concentration of 400 µg/mL, the degree of inhibition was 62.37 ± 1.24%.

The obtained results confirm that the investigated samples have a pronounced ability to prevent thermal denaturation of albumin, indicating their potential anti-inflammatory activity and promising for biomedical applications [73].

The hemostatic activity of PVA/KAO_0.25_/CS/NaCMC and PVA/KAO_0.5_/CS/NaCMC samples was evaluated in comparison with an intact control group and the industrial hemostatic drug “Statin”. The main parameters characterizing the effectiveness of the substances studied were the average time to stop bleeding and the volume of blood loss in laboratory rats (Figure 7D,E). It was established that in the animals of the control group, the average time to stop bleeding was 102.33 ± 5.13 s (Figure 7D). The use of the PVA/KAO_0.25_/CS/NaCMC sample at a dose of 100 mg significantly reduced the time to stop bleeding to 59.67 ± 1.53 s. At the same time, the drug “Statin” demonstrated the most pronounced reduction in time—to 35.33 ± 5.51 s (Figure 7D). It was found that the use of the PVA/KAO_0.5_/CS/NaCMC sample significantly reduced the time to stop bleeding. At a dose of 50 mg, this indicator was 81.67 ± 10.02 s, and at a dose of 100 mg, it was 62.0 ± 6.56 s (Figure 7D). The hemostatic time in the liver laceration models of rats was from 99 to 134 s of CS/kaolin composite microspheres with different mass ratios [74]. The PVA/ γ-chitosan/kaolin nanofiber wound dressings show blood clotting times ranging from 51 to 76 s [75].

An investigation of blood loss in a model of simulated liver damage showed that in the intact control group it was 2482.07 ± 198.53 mg (Figure 7E). The use of the PVA/KAO_0.25_/CS/NaCMC sample at a dose of 100 mg reduced blood loss to 1485.37 ± 79.88 mg, and when “Statin” was used, it amounted to 829.83 ± 73.86 mg. Also, in the PVA/KAO_0.5_/CS/NaCMC sample at a dose of 50 mg, blood loss was 1545.47 ± 51.13 mg, and at a dose of 100 mg, it was 1428.73 ± 8.23 mg (Figure 7E).

Thus, the samples have pronounced hemostatic activity, significantly reducing the time to stop bleeding in rats and the volume of blood loss compared with the intact control group. The most pronounced effect was observed at a dose of 100 mg, which indicates the dose-dependent nature of the hemostatic action.

#### 3.7.2. Antimicrobial Activity

**Figure 8:**
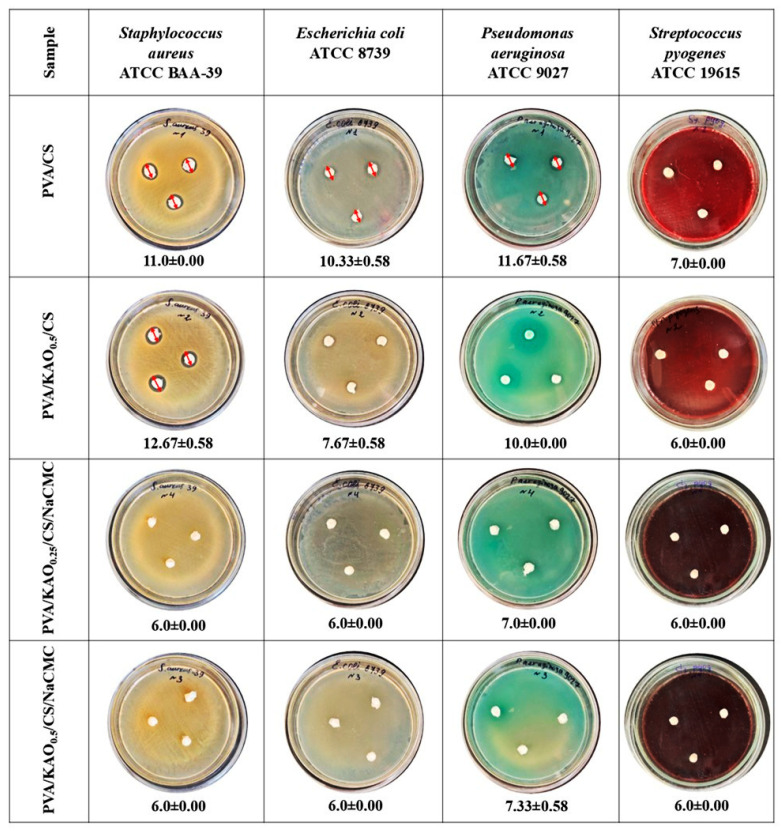
Results of antimicrobial activities of PVA/CS, PVA/KAO_0.5_/CS, PVA/KAO_0.25_/CS/NaCMC, and PVA/KAO_0.5_/CS/NaCMC hydrogels by diffusion method against *Staphylococcus aureus* АТСС ВАА-39, *Escherichia coli* АТСС 8739, *Pseudomonas aeruginosa* АТСС 9027, and *Streptococcus pyogenes* АТСС 19615 test strains. Zone of growth inhibition, mm ± StD.

The antimicrobial activities of PVA/CS, PVA/KAO_0.5_/CS, PVA/KAO_0.25_/CS/NaCMC, and PVA/KAO_0.5_/CS/NaCMC were tested by diffusion test against Gram-positive bacteria *Staphylococcus aureus* ATCC BAA-39 and *Streptococcus pyogenes* ATCC 19615 and Gram-negative bacteria *Pseudomonas aeruginosa* ATCC 9027 and *Escherichia coli* ATCC 8739. The results of the diffusion test are shown in Figure 8.

The PVA/CS hydrogel sample also showed good activity, especially against *Pseudomonas aeruginosa*, where the zone of inhibition was 11.67 ± 0.58 mm, and against *Staphylococcus aureus*, where the zone of inhibition was 11.0 ± 0.00 mm. This confirms that chitosan has antimicrobial properties [76]. The PVA/KAO_0.5_/CS hydrogel showed the highest antimicrobial activity against most strains where the zones of inhibition were 12.67 ± 0.58 mm against *Staphylococcus aureus*, 10.0 ± 0.00 mm against *Pseudomonas aeruginosa*, and 7.67 ± 0.58 against *Escherichia coli*. The performance of this sample is significantly higher than other samples, and this confirms that this sample has the best synergistic effect of kaolinite at 0.5 weight concentration. In hydrogels with NaCMC, PVA/KAO_0.25_/CS/NaCMC and PVA/KAO_0.5_/CS/NaCMC, a negligible zone of inhibition of 6.0 ± 0.00 mm was observed, indicating no significant antimicrobial activity. The results confirm the promising potential of the prepared hydrogels in terms of antimicrobial activity.

## 4. Conclusions

The synthesized hydrogels PVA/KAO_0.25_/CS, PVA/KAO_0.5_/CS, PVA/KAO_0.25_/CS/NaCMC, and PVA/KAO_0.5_/CS/NaCMC were prepared by a freeze–thaw method. The physicochemical properties of the hydrogels were examined in terms of thermal stability, morphology and structure, porosity, swelling, gel fraction, and mechanical properties. The mechanical tensile strength value observed for the PVA/KAO/CS sample indicates that increasing kaolinite concentration improves the tensile strength and elasticity of the material. The obtained hydrogels showed hemolysis of about 2.5% and about 3% for PVA/KAO_0.25_/CS/NaCMC, which is considered a safe level according to ASTM, and the % of inhibition of protein denaturation meets the requirements and is about 60–70%.

It has been established that the PVA/KAO_0.25_/CS/NaCMC and PVA/KAO_0.5_/CS/NaCMC samples have pronounced hemostatic activity, significantly reducing the time to stop bleeding and the volume of blood loss in rats compared with the intact group. The effect is dose-dependent, with the best results achieved at a dose of 100 mg.

Also, the resulting hydrogels exhibited safe hemolytic activity and antimicrobial activity against Gram-positive bacteria *Staphylococcus aureus* ATCC BAA-39 and *Streptococcus pyogenes* ATCC 19615 and Gram-negative bacteria *Pseudomonas aeruginosa* ATCC 9027 and *Escherichia coli* ATCC 8739. The PVA/KAO_0.5_/CS hydrogel showed the highest antimicrobial activity against most strains.

The obtained results confirm that the synthesized hydrogels have a pronounced ability to prevent thermal denaturation of albumin, indicating their potential anti-inflammatory activity and antimicrobial activity, both promising biomedical applications.

The hydrogel PVA/KAO_0.5_/CS/NaCMC is the most promising in terms of its biomedical properties and can be used as a hemostatic agent for skin wounds after additional in vitro and in vivo studies.

## Data Availability

The data presented in this study are available on request from the corresponding author.

## Abbreviations

The following abbreviations are used in this manuscript:

PVA	polyvinyl alcohol
KAO	kaolinite
CS	chitosan
